# Supplementary figures and images for: Neural signatures of arbitration between Pavlovian and instrumental action selection
Source: PLoS Comput Biol. 2021 Feb 10;17(2):e1008553. doi: 10.1371/journal.pcbi.1008553 (PMC7901778; doi:10.1371/journal.pcbi.1008553)

**Guitart-Masip et al. (2012)**

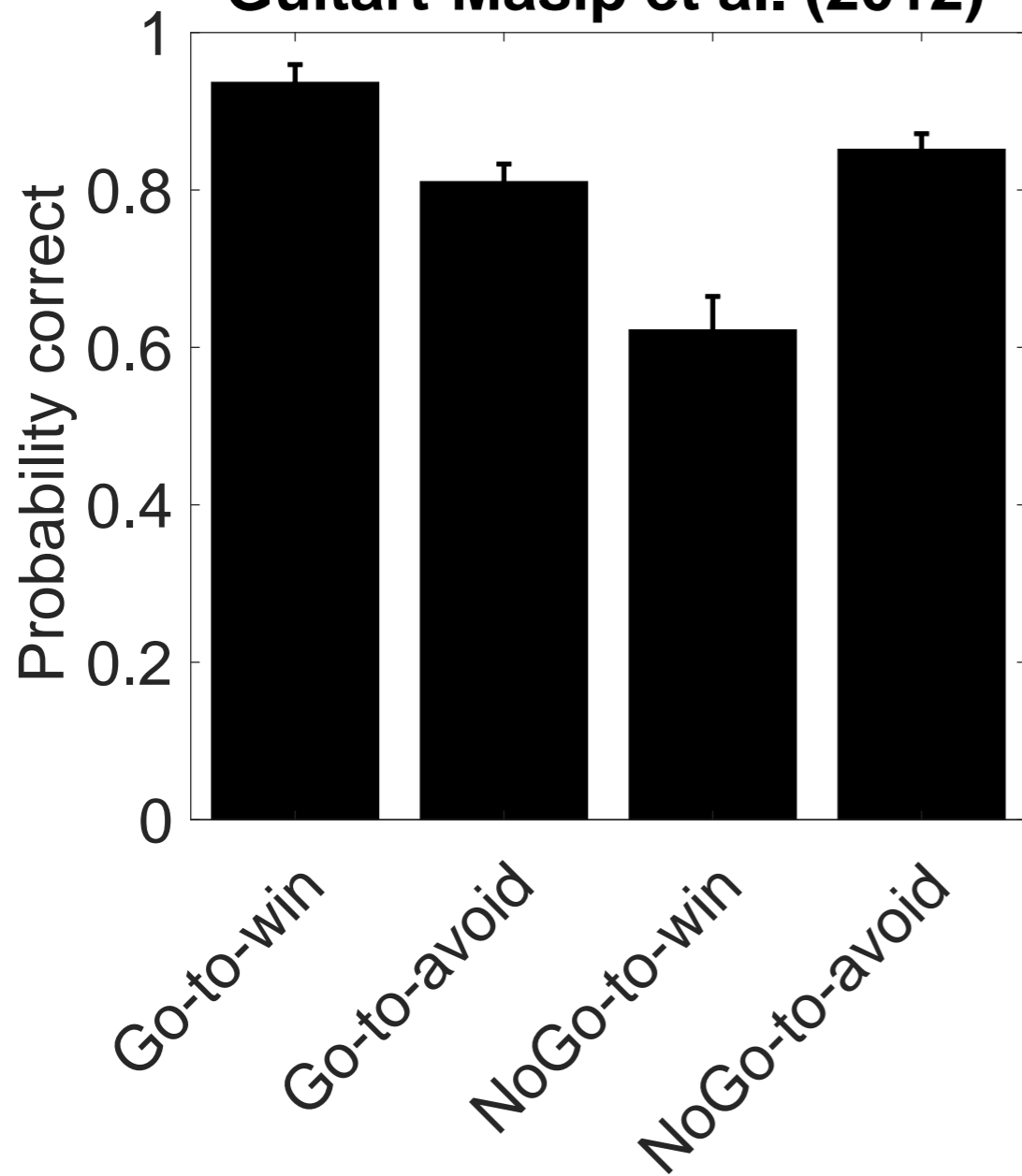

**Model simulation**

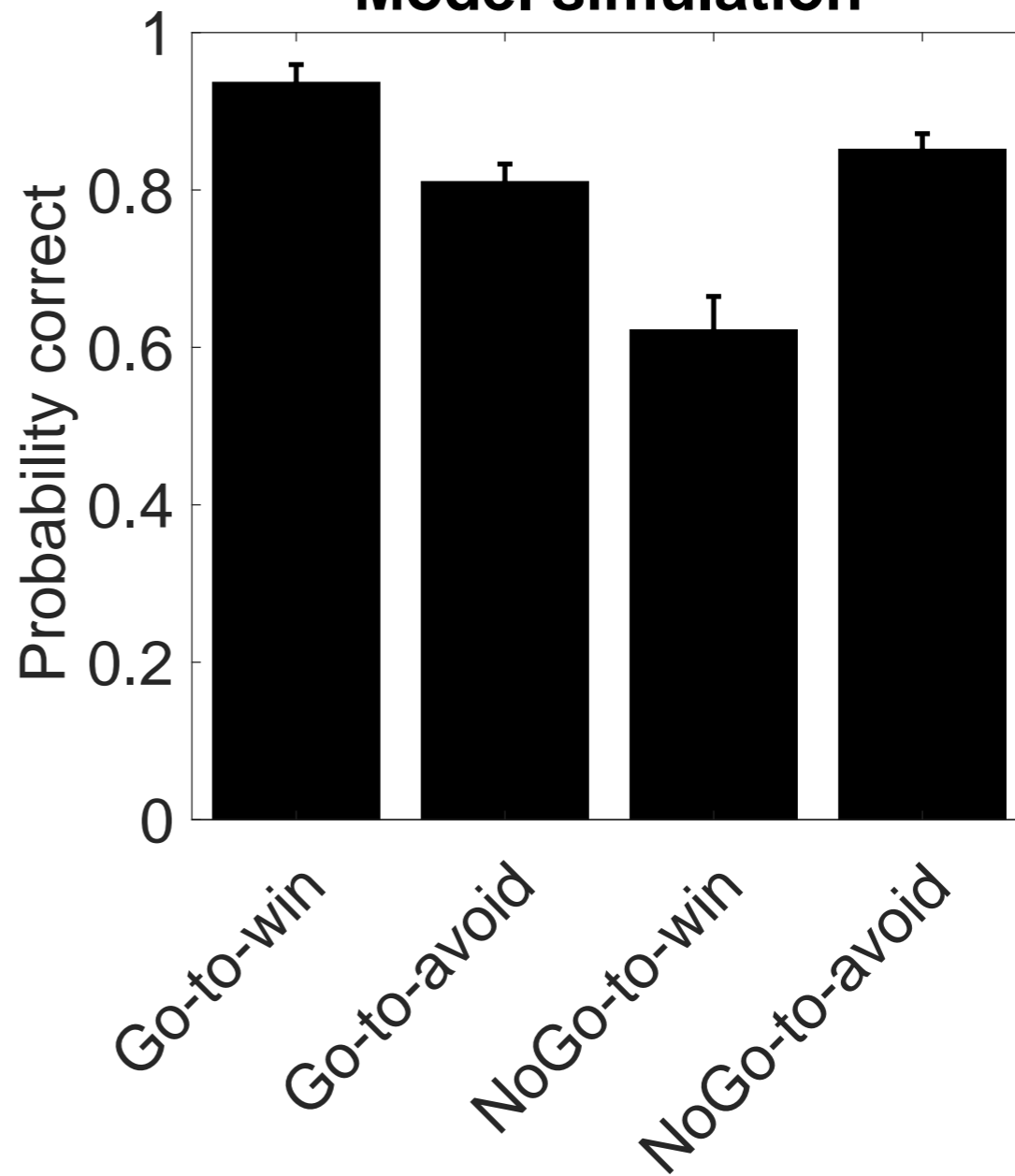

Supplement: S1 Fig — (Left) Data from Guitart-Masip et al. (2012). (Right) Simulations of the adaptive Bayesian model, using parameters fitted to the data. Error bars show standard error of the mean. (PDF) [file pcbi.1008553.s001.pdf]

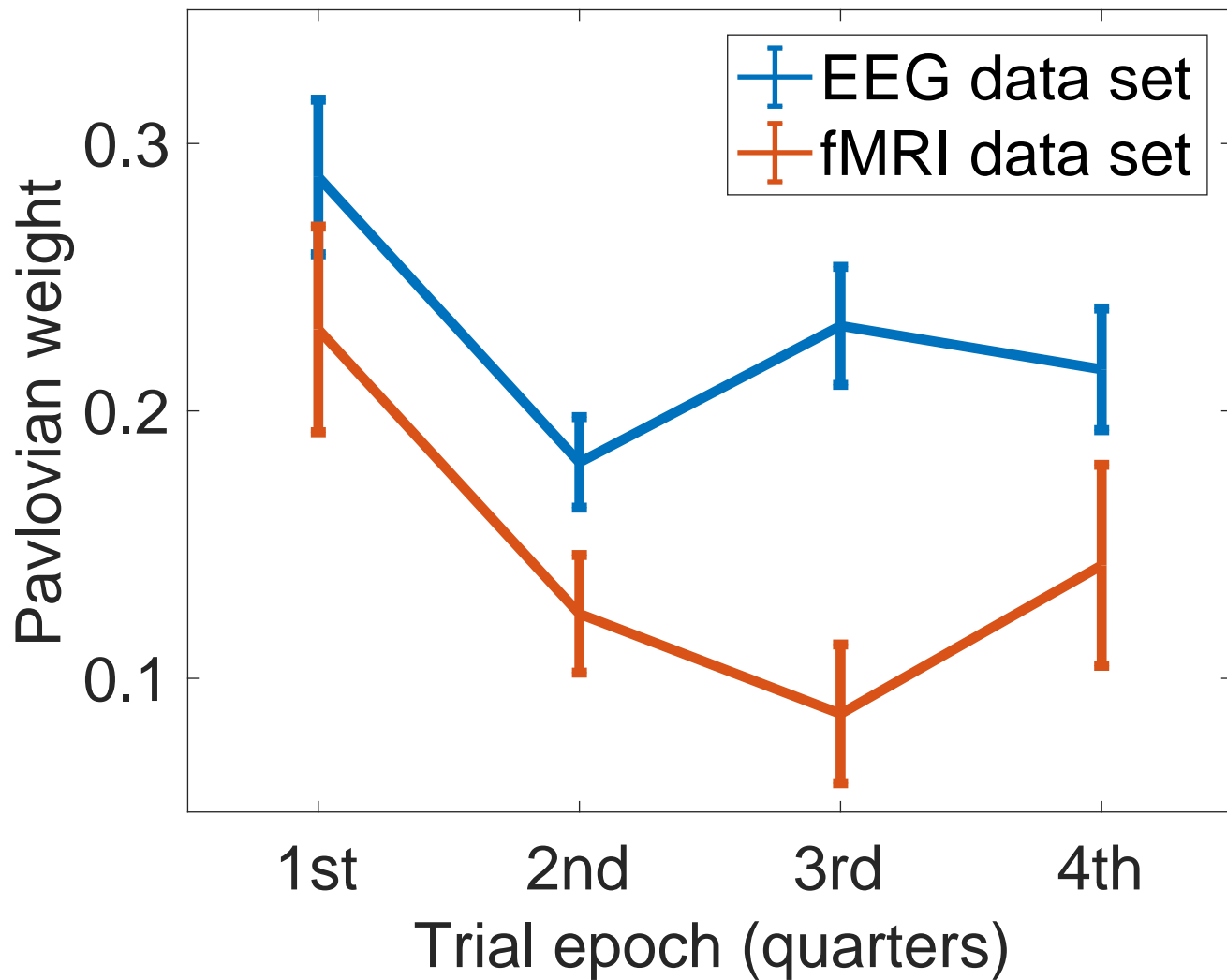

Supplement: S2 Fig — The weight variable w is plotted across trial epochs, broken into quarters (note that the data sets have different numbers of trials). Error bars show standard error of the mean. (PDF) [file pcbi.1008553.s002.pdf]

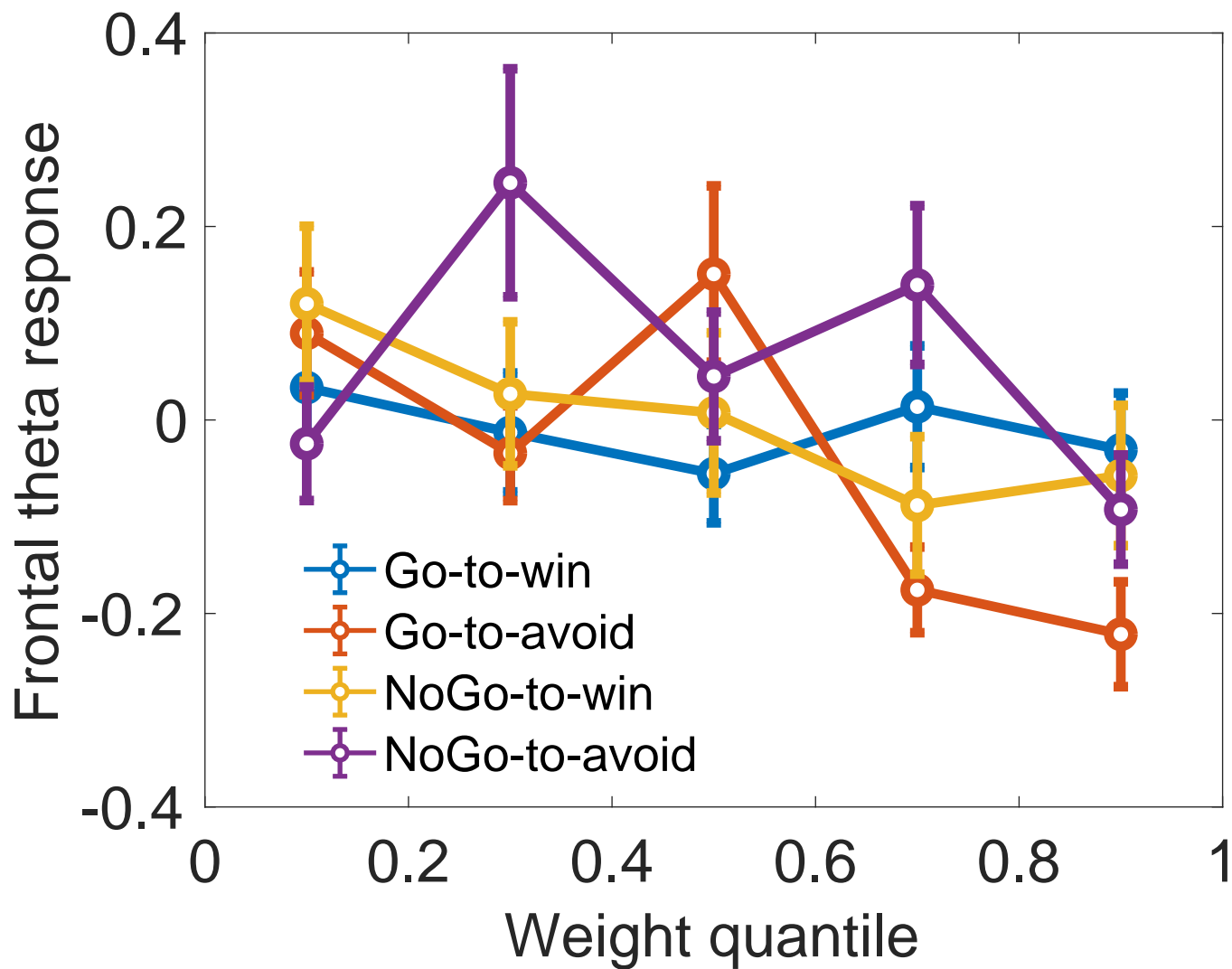

Supplement: S3 Fig — Midfrontal theta power (z-scored within subject) as a function of Pavlovian weight quantile, separated by stimulus condition. Error bars show standard error of the mean. (PDF) [file pcbi.1008553.s003.pdf]

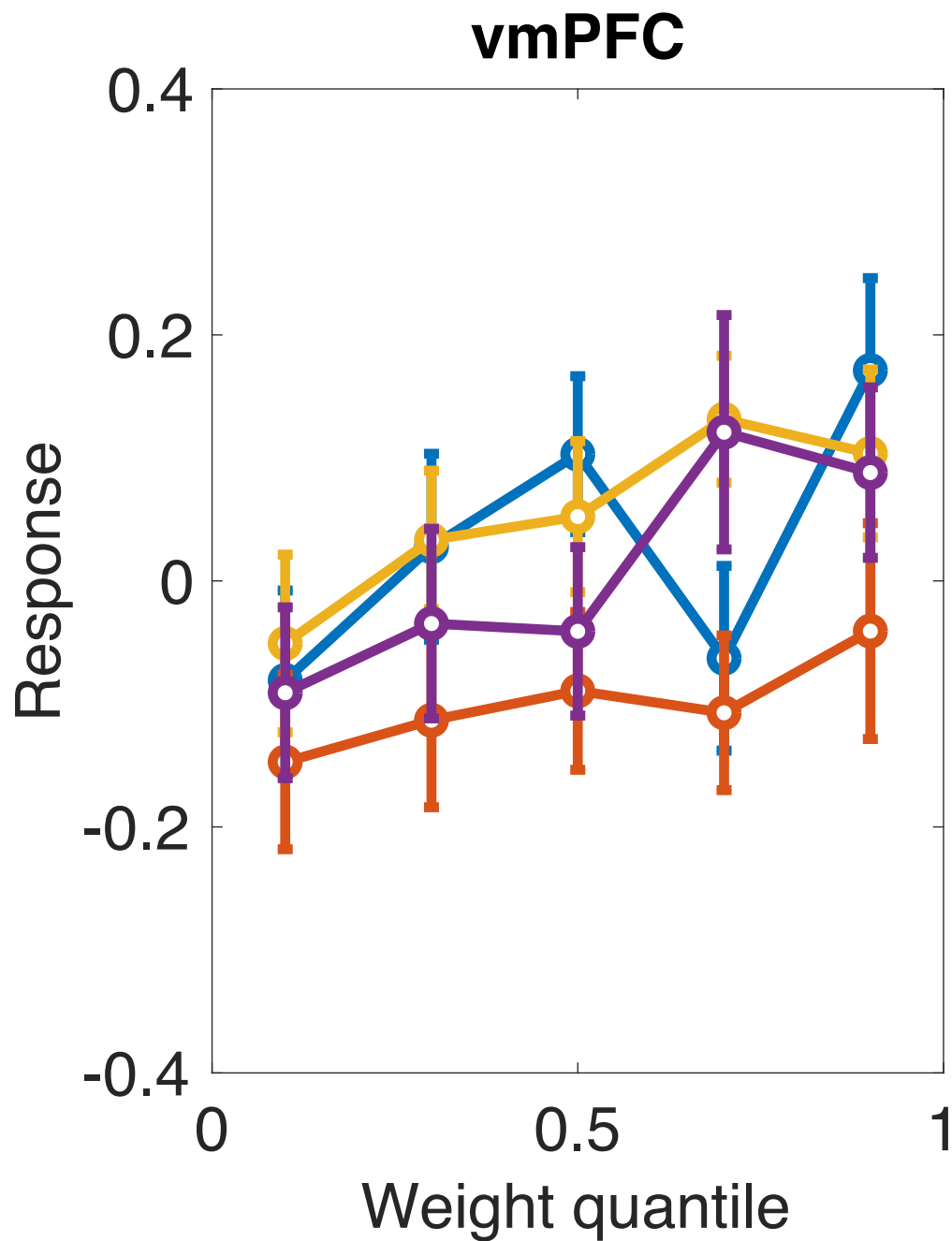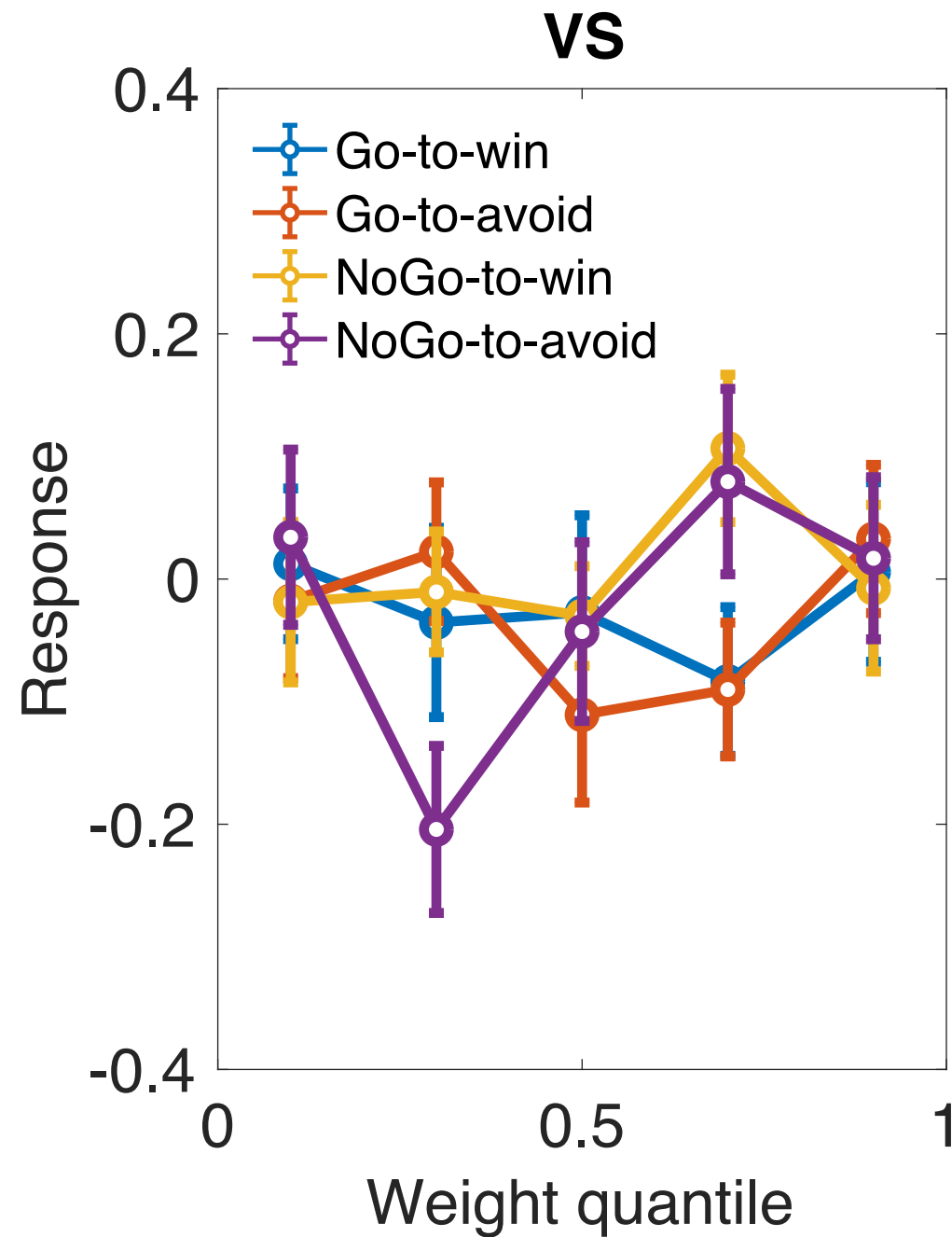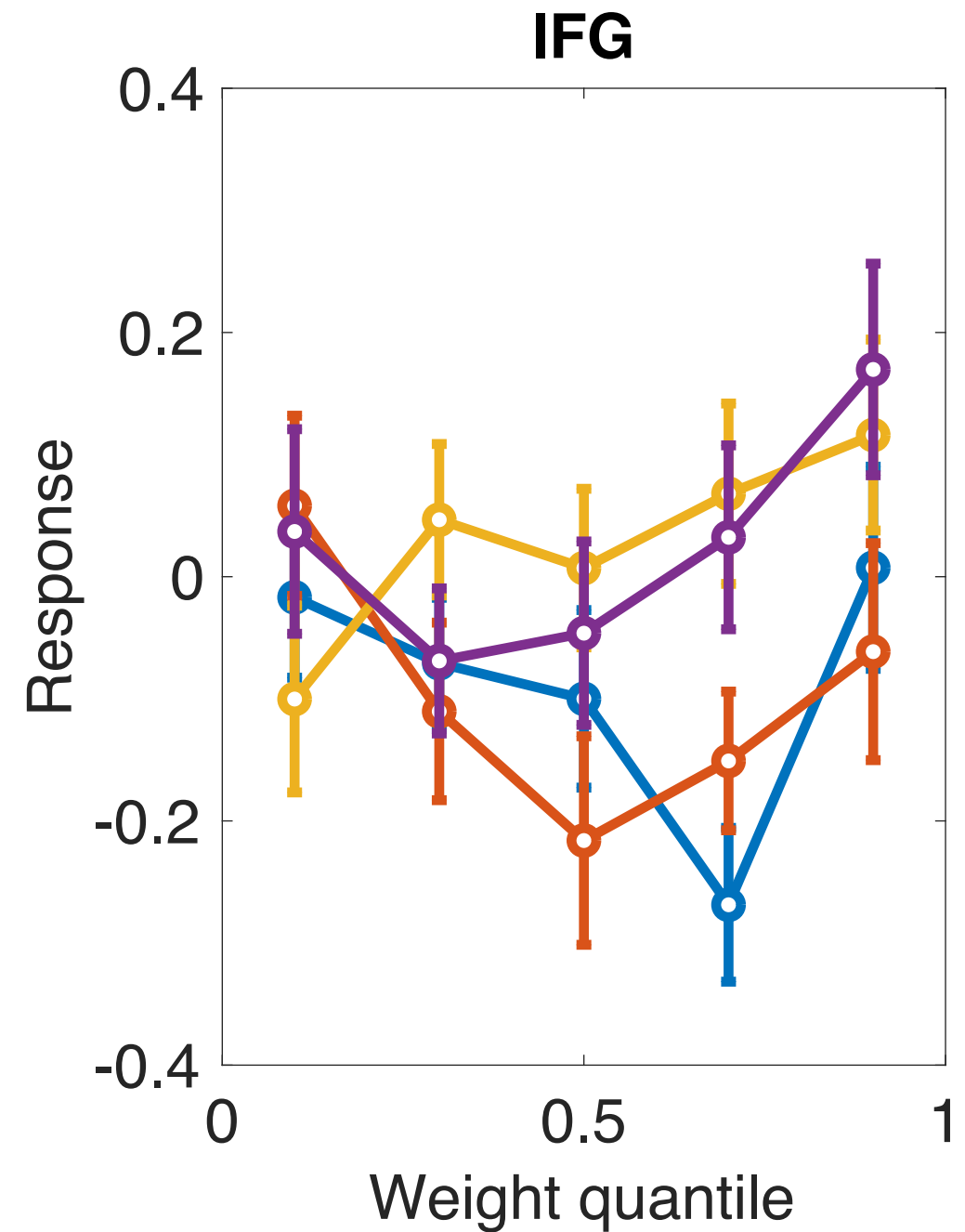

Supplement: S4 Fig — BOLD response amplitude (z-scored within subject) as a function of Pavlovian weight quantile, separated by stimulus condition. Left: ventromedial prefrontal cortex. Middle: ventral striatum. Right: inferior frontal gyrus. Error bars show standard error of the mean. (PDF) [file pcbi.1008553.s004.pdf]

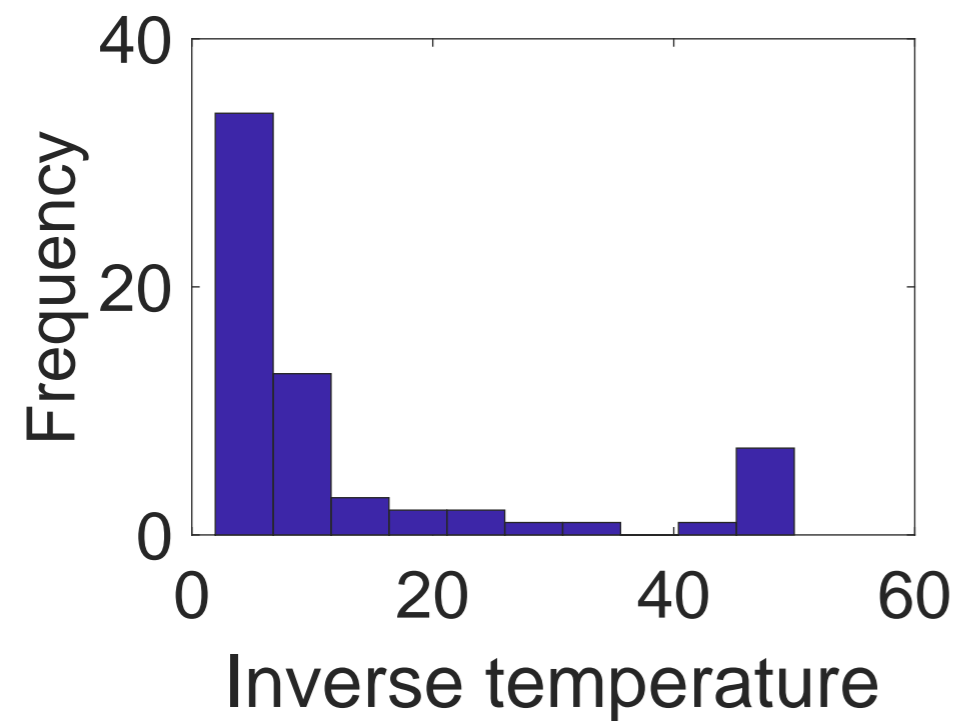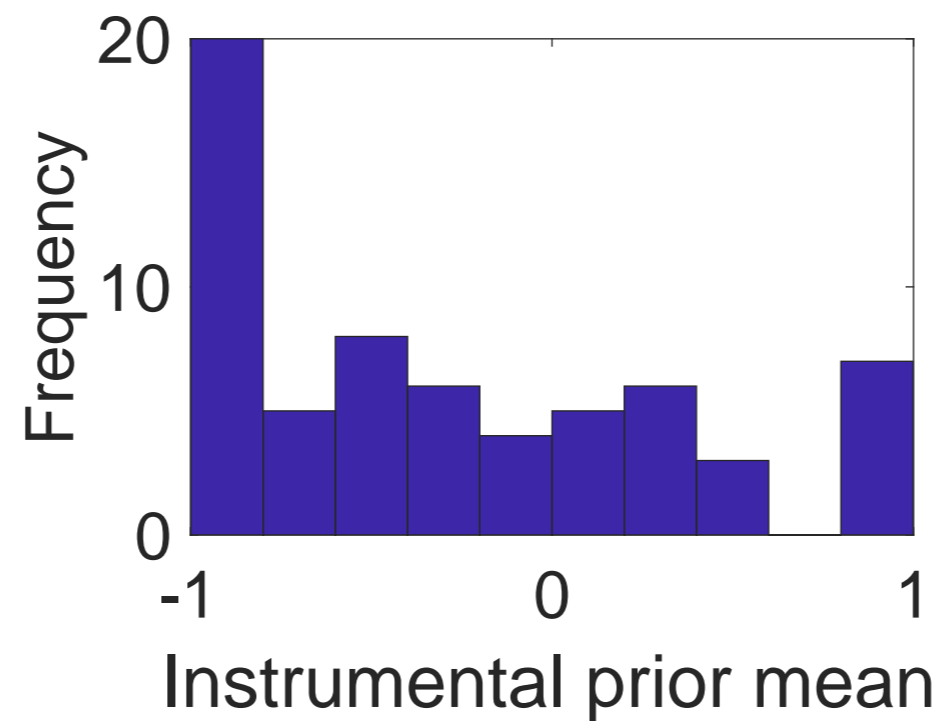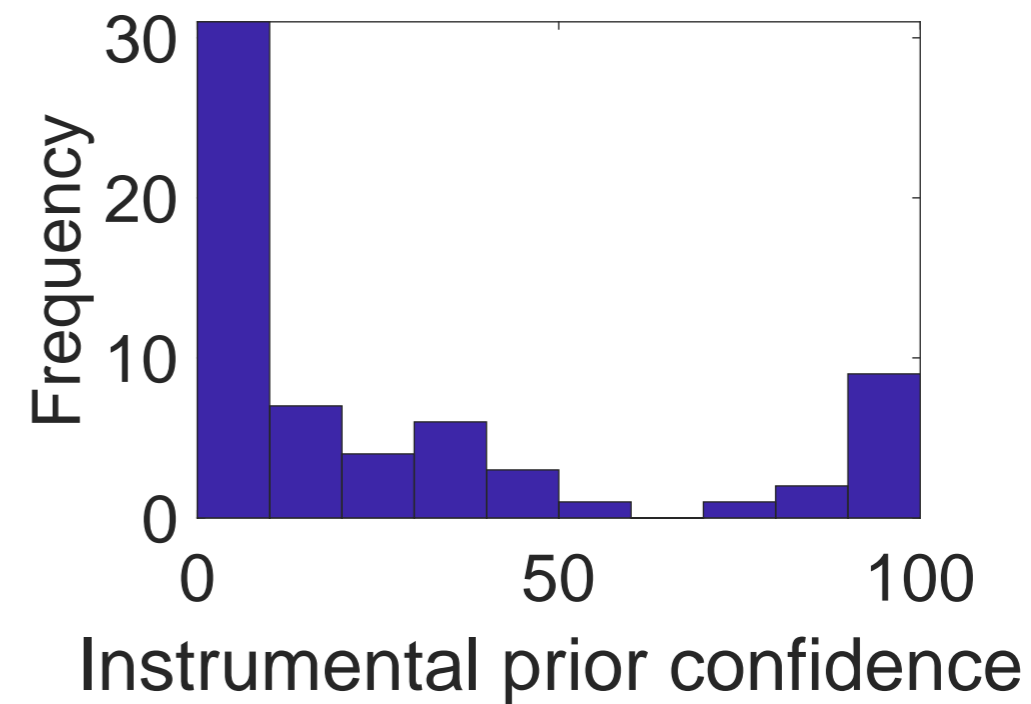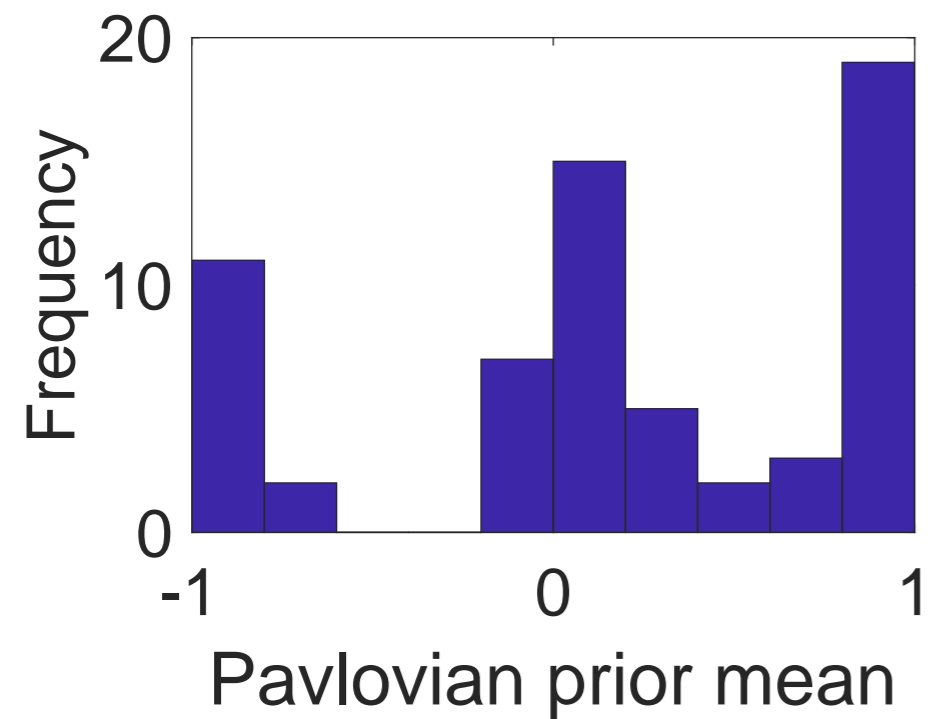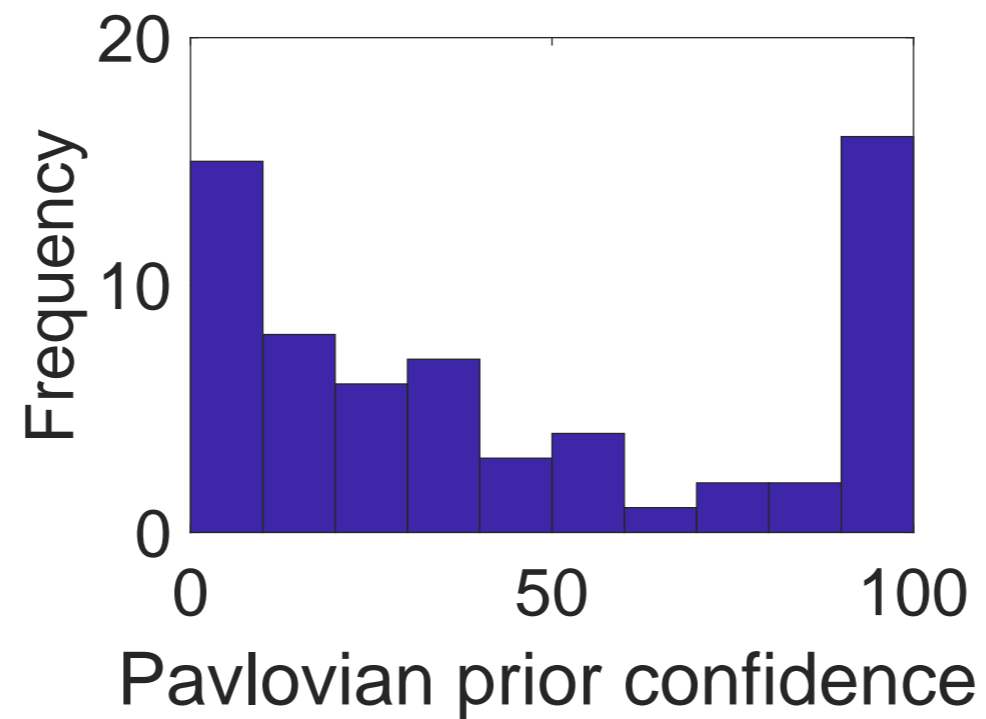

Supplement: S5 Fig — Estimates were aggregated across the EEG and fMRI data sets. (PDF) [file pcbi.1008553.s005.pdf]
