# Supplementary material for: Neural signatures of arbitration between Pavlovian and instrumental action selection
Source: PLoS Comput Biol. 2021 Feb 10;17(2):e1008553. doi: 10.1371/journal.pcbi.1008553 (PMC7901778; doi:10.1371/journal.pcbi.1008553)
Supplement: Attachment — Submitted filename: response.pdf [file pcbi.1008553.s006.pdf]

Dear Dr. Marinazzo,

Thank you for communicating these reviews, which have helped us improve the manuscript. Below we respond to each comment. Here is a summary of the major changes:

- We have carried out additional predictive checks to ensure that our model is fitting the data well.
- We have confirmed parameter and model recoverability.
- We have clarified several points of confusion that came up in the reviews.

As you will see, in responding to these comments we have tried to balance the need to comprehensively address the reviewers' concerns with the need to maintain expository clarity and focus. Several of the issues raised by the reviewers are, in our view, important but also outside the scope of the questions that we are trying to answer here.

Best wishes,

Sam Gershman

Reviewer #1

The manuscript offers a refreshing new way to look at PIT from a computational perspective. The insights that uncertainty controls arbitration between two models – a Pavlovian and a Bayesian – relies on important literature and applies it to PIT in an elegant way.

We thank the reviewer for this positive evaluation. We would like to note, however, that we are not modeling PIT (at least as it is traditionally conceived). This point is clarified below.

The main body of work that the authors use to motivate their model is reference [19]. They sketch two implications of the model, as follows:
• "First, the Bayesian arbitration mechanism preferentially allocates control to the Pavlovian process initially, when there are less data and hence less support for the more flexible model. This is broadly consistent with the finding that the Pavlovian bias on instrumental responding declines with the amount of instrumental training [19]." – I understand this to mean: MORE instrumental training, LESS PIT
• Second, this initial preference should be stronger in relatively less controllable environments, where little predictive power is gained by conditionalizing predictions on action. Accordingly, Pavlovian bias increases with the amount of Pavlovian training [19]." I understand this to mean: MORE Pavlovian training, MORE PIT

I may well be misunderstanding something, and therefore stand to be corrected, but reference [19] (and indeed my knowledge of the literature on which it is based) appear to say something very different: the exact set-up of the conditioning phases (prior to the PIT phase) greatly influence the pattern found; so we ignore them in our peril. The findings agree with the authors' interpretation only for a subset of experimental set-ups, but contradict them in many others.

Reference 19 makes 3 contributions. It provides a subjective review of the literature; a meta analysis; and new empirical data.

Quoting from the review: "Greater amounts of instrumental training facilitate PIT (Holland, 2004), leading some to consider that habitual responses are more susceptible to the general motivating influence of CSs (Holland, 2004, Yin and Knowlton, 2006; see also Dickinson and Balleine, 2001). " I understand this to mean: MORE instrumental training, MORE PIT – the opposite of the authors' interpretation.

From the empirical work: "Extensive Pavlovian conditioning produced more Pavlovian magazine visits and weaker PIT than moderate Pavlovian conditioning (Experiment 1)". I understand this to mean: MORE Pavlovian training, LESS PIT – again, the opposite of the authors' interpretation.

From the meta-analysis: "The amount of instrumental training clearly influences PIT scores in non-selective transfer studies and to some extent in selective transfer studies. The precise relationship between instrumental training and PIT scores furthermore depends on the order of the instrumental and Pavlovian conditioning phases. More instrumental training facilitates PIT when Pavlovian conditioning precedes instrumental training, but appears to be detrimental to PIT when the order of the two phases is reversed." Here, half the studies agree with the authors' interpretation, but the other half directly contradict it.

From the meta-analysis: "There were no relationships between PIT scores and amounts of Pavlovian conditioning for groups in non-selective PIT studies. …" In "Selective PIT studies… When Pavlovian conditioning preceded instrumental training, there was a clear negative relationship between PIT scores and the amount of Pavlovian conditioning… However, when Pavlovian conditioning followed instrumental training, there was a positive relationship between PIT scores and the amount of Pavlovian conditioning for CS-different… but not for CS-same". My understanding here is that non-selective studies contradict the authors' interpretation; half of the selective studies contradict it (MORE Pavlovian conditioning, LESS PIT); and some of the other half of selective studies agree with it.

For the model to be useful for the community of PIT researchers, the proposed model need to exhibit some of the agreed patterns in that literature. Of course, it's expected that

the model will make some novel predictions; but when it contradicts existing patterns, this needs to be very clearly spelled out. At present, as far as I see, the crucial reference the authors used highlights important differences between the experimental set-ups that give rise to different patterns of the relationship between training and PIT, which the current model does not have within it a mechanism to explain. It would be important, for example, to relate the key variable of interest here – controllability – to the order of training (Pavlovian vs. Instrumental first). I would love to have the author's response to this query, because I do agree with the huge potential of their approach.

We thank the reviewer bringing up these issues, which we had glossed over in the Introduction. Upon reflection, we feel that it would be a mistake to try to tackle the complex set of PIT phenomena in our manuscript, for several reasons. First, it significantly broadens the scope of the manuscript beyond what we were trying to accomplish, which is to understand the neural computations underlying behavior in the Go/NoGo task. We would like to emphasize that our immediate goal is not to build a model that is useful for the community of PIT researchers, but rather to test a model that was developed to explain a specific task. Second, it's not clear that PIT and Go/NoGo tasks are measuring the same thing. As far as we know, no one has done a systematic comparison (for example, we don't know whether PIT and Go/NoGo effects are correlated across individuals). It's entirely possible that these tasks are measuring different (but possibly overlapping) cognitive mechanisms.

All that being said, we do think that there is potential for a model of this sort to explain the PIT phenomena described above. One of the critical issues, in our view, is the stimulus-specificity of controllability inferences in the Bayesian model. We have assumed that controllability is a "global" property that generalizes across stimuli. In the Dorfman and Gershman variant of the Go/NoGo task, in which controllability was explicitly manipulated, care was taken to intersperse the stimuli (which is also the case for the data analyzed in our manuscript). This was done to ensure that subjects did not infer "local" controllability (i.e., specific to particular stimuli). The PIT paradigm, in contrast, involves a temporal separation of Pavlovian and instrumental training phases, which may encourage local inference. As a consequence, we would expect different behavior in the two paradigms. From an experimental perspective, we could test this hypothesis by temporally separating the stimuli in the Dorfman and Gershman Go/NoGo task. From a theoretical perspective, addressing this issue will require an extension of the model that allows it to determine the stimulus-specificity of controllability. It's unclear whether such an account could explain order effects in PIT, as well as differences between selective and non-selective studies; we would need to undertake a substantively new modeling project to answer these questions.

Both of these directions seem worth pursuing, but they would take us far afield from the goals of this manuscript. We therefore felt that the simplest solution is to remove the discussion of PIT from the Introduction. No other part of the manuscript depends on claims about PIT. We now mention it briefly as an open challenge at the end of the Discussion (p. 10):

“Third, while our work is partly motivated by studies of Pavlovian-instrumental transfer [Holmes et al., 2010], it is still an open empirical question to what extent that phenomenon is related to Pavlovian biases in Go/NoGo performance. It is also an open theoretical question to what extent the kind of Bayesian arbitration model proposed here can provide a comprehensive account of Pavlovian-instrumental transfer.”

Reviewer #2

In general, the paper is well-written with a clear research question and a dedicated computational model that is being tested. Model comparison with degenerate version of the model and a previously published RL model is done with appropriate methods. I also like the idea of recycling and reanalyzing older data sets to uncover novel aspects of already established tasks. However, in the current paper, there many elements missing that one would want to see in a computational modeling study. This dampens my enthusiasm for this paper considerably at this point.

Model simulation and parameter recovery. It would be good to know that the proposed model is able to recover true parameters value during MLE estimation (Why was the estimation not done in a hierarchical manner?). In addition, it would be interesting to see, how different Pavlovian weights change the model-free signature of hte behavioral data (e.g. the interaction of response (Go/NoGo) and valence (win / neutral/ loss) that was reported in the original publications.

We appreciate this suggestion. We have added a paragraph to the Methods detailing recoverability:

“We verified that parameters of the adaptive Bayesian model are reasonably recoverable, by simulating the experimental design used in [Cavanagh et al., 2013], with the same number of subjects, and then fitting the simulated data. Overall, the correlation between true and recovered parameters was r=0.59 (p<0.0001). Two parameters exhibited relatively poor recoverability, with r=0.32 for the prior mean of the instrumental values, and r=0.33 for the prior confidence of the Pavlovian values, but we do not make any claims about these parameters in the paper. In addition to parameter recoverability, we found good model recoverability: the protected exceedance probability assigned to the adaptive Bayesian model was close to 1.”

We chose to use the same fitting methods as described in Dorfman & Gershman (2019), which is why the models were not fit hierarchically.

We did not find any parameter correlations with model-free measures of Pavlovian bias (as defined in Cavanagh et al., 2013). Note that our goal in this paper is not to explain individual differences but to explain variations *within* an individual session.

Model accuracy and Posterior predictive checks. We are presented with evidence that the flexible Bayesian learner fits the data best among the competing models, bug we don't actually see, if this model is able to generate data from the fitted parameters that are commensurate of the experimental data. For instance, how accurate is the model fitting to the original experimental data and can the model replicate the finding in the original publication of an interaction of Go/NoGo and Valence?

This is a good suggestion. We have now run simulations of the model to show that it captures the original Guitart-Masip et al. (2012) accuracy effects (see Figure S1).

Analysis of the fitted parameters. We never get to see the distribution of the model parameters between different participants and whether they are related behavioral performance of the imaging data.

We have now added Figure S5, which summarizes the parameter distributions. As mentioned above, we did not observe any systematic relationships between these parameters and behavioral measures. We chose to put this information in the supplement in order to maintain a focus on the within-subject variability of neural and behavioral measures that we are trying to explain.

Inconsistencies between DFs and participant number. There is an apparent inconsistency between the degrees of freedom in paired t-tests (EEG df=31, fMRI df=28) and the number participants in the two samples (EEG n=30, fMRI n=47). Were there any subjects excluded from the modeling and, if yes, for what reasons? Please resolves this inconsistency.

Sorry for this confusion. The data set reported in Guitart-Masip et al. (2012) contained 47 subjects, but only 30 with fMRI. Also, we realized there was a typo; there were 34 subjects in the EEG data set. We've now corrected this in the Methods.

1st level fMRI GLM. I am confused about the 1st level GLM in the fMRI data set. There were trial-specific regressors and in addition parametric regressors for Go/NoGo and Outcome. The latter models the experimental variance due to these two factors that is common across trials, which leaves the trial-specific regressors with the residual variance that is not explained by these common factorial regressors. How is it then possible that the ROI analysis based on the trial-specific beta images is still showing effects of valence and behavioral response.

The Go/NoGo modulator is based on the subject's *response*, but the Go/NoGo analyses in the main text are based on the *stimulus type* (experimental condition). We included the response regressor to remove purely response-related activity, which allowed us to better isolate responses to the stimulus type. Similarly, outcome type is distinct from valence (a property of

the stimulus type). This allows us to control for the effect of outcome that might bleed into the effect of stimulus type, as we explain in the Methods.

Incomplete analysis of the data. The authors correctly mention the caveat that they only analyzed responses to cue presentation, but not following the outcome leading to value updates in the model. However, they hav the data at hand to look at this question and going the extra mile would give a comprehensive picture of the neural computations associated with controllability.

This is a good recommendation in principle, but in practice there are several issues that limit the usefulness of outcome-locked activity in these data sets. The EEG data set was actually originally collected to look at outcome-locked activity, but it turned out that there were too few trials in some conditions. A similar issue occurs with the fMRI data set. Basically, the problem is that looking at outcome-locked activity requires separation by outcome type, which means that there are just too few trials. Our experience with these tasks suggests that we would need them to be 3x longer.

It's also worth keeping in mind that our primary hypothesis concerned stimulus-locked activity: we wanted to identify neural correlates of the arbitration process, which is likely invoked at decision time. Outcome-locked activity doesn't really help us address this question.

Figure Permission. Figure 1b is taken directly from the original Dorfman & Gershman, NCOMMS paper, which should be cited in the Figure legend.

We now acknowledge the provenance of this figure in the caption.

Reviewer #3

Overall, the underlying theory is very interesting and elegant but has already been published in Dorfman & Gershman (2018). The presented re-analyses of behavior, EEG and fMRI data from two previous studies are a bit superficial in the current state. A number of potential confounds have been neglected (see detailed suggestions below). Unless much more thorough analyses are presented and drastic improvements of the manuscript are made, I have the impression that not much can be learned from the paper compared to the two previous studies from which the datasets were taken, and which already showed clear neural correlates of Pavlovian influence over behavior and its modulation/suppression.

The original studies did not study arbitration at all. The models reported in those studies had no way of identifying trial-by-trial variations in Pavlovian influence on action selection. We consider this to be a novel and significant contribution. It's also notable that we were able to apply a

model, without modification, to data that were not originally designed to test the model: this demonstrates the generality of the model introduced by Dorfman and Gershman.

Detailed comments

The authors found an initially positive Go bias even in the Avoid condition in resp. 76% and 63% of the subjects of the datasets. This seems contradictory with the hypothesis that Pavlovian biases should intrinsically favor Go for Win and NoGo for Avoid (Huys et al., Disentangling the roles of approach, activation and valence in instrumental and pavlovian responding. PLoS Comput Biol 7, e1002028 (2011).). How do the authors reconcile their observation with the underlying theoretical hypothesis?

A consistent finding across these studies is that there is a generic Go bias in addition to a learned state-specific Pavlovian bias (which is either towards Go or NoGo depending on reward vs. punishment conditions). So our finding is completely consistent with the earlier studies. The main difference lies in how we explain the generic Go bias. Instead of modeling it as an additional parameter that enters into the decision value, we modeled it in terms of the prior over values (i.e., when the prior mean over the Pavlovian value is positive, a Go bias will be present even at the beginning of training). We now note this point in the Results section when we report the analysis (p. 6).

The tested model space is too small. Why not comparing to a non-Bayesian RL model with annealed learning rate, similar to the Bayesian model, so as to assess the specific role of this annealing process? It would also be interesting to compare the model to a fixed amplitude change model rather than based on RPE amplitude (to assess the variability of magnitude changes), and to compare the model to a random walk model (to assess the consistency of direction changes).

We wish to make a plea for the importance of expository clarity, which would be ill-served by the addition of these models. We chose our models because they exemplify key hypotheses about the nature of cognitive processes in the Go/NoGo task. They are not meant to exhaust the space of models, but rather to distill it. If our goal is to use models as explanatory tools, we should strive to present a small number of models that clearly delineate qualitatively different accounts of the data. This is what we have done in selecting the 3 models reported in the paper.

It's also important to keep in mind that what we designated the "RL model" was already subjected to stringent model comparison in previous analyses of the fMRI data set (see Guitart-Masip et al., 2012). We are building on those results.

We don't mean to be dismissive of these alternative models, which might be useful for other purposes. We just don't see why they're relevant for addressing the question of arbitration. For example, does it matter how exactly the learning rate changes over time? Undoubtedly we do not have a precise understanding of learning rate dynamics; the Bayesian model is simply an

approximation. So finding that we might do better or worse in matching behavior by modifying these dynamics doesn't really speak to our core hypotheses. Similarly, it's unlikely that we have precisely captured the magnitude-dependence of learning, but our core hypothesis does not concern this issue, so showing that we can do better or worse in capturing behavior is tangential to our goals.

The authors systematically plot variables of interest against weight quantile (e.g., Figs. 2, 3). This is interesting but not sufficient to evaluate the temporal evolution of these variables. In Dorfman & Gershman (2018; Fig. 6), the Go bias decreases rapidly in less than 10 trials, and then remains flat during the rest of the experiment. Is this also the case here? Is it also the case for the Pavlovian weight and for the variables of interest plotted in Figs. 2 and 3? Figure S1 shows the evolution of the Pavlovian weight through time. However, it is important to plot the trial-by-trial evolution, and not just for different quarters of trials. Moreover, it is important to show distinct plots for different conditions and different groups of subjects (see next paragraph). Finally, because the Go bias and Pavlovian weights are expected to change a lot during early experiment and then to remain nearly flat, it is important to verify that correlations with the model's Pavlovian weight still hold when only considering the second half of each condition block.

Figure S1 shows that the Pavlovian weight declines across trials, though it's unclear whether it's as fast as in Dorfman & Gershman. It's not obvious to us why this matters, though; we haven't made any strong claim about the speed with which the weight declines.

The reason we plotted it in epochs rather than trial-by-trial is because there was a large amount of variability across subjects, and fewer subjects in these studies compared to Dorfman & Gershman. So some trial averaging was helpful in revealing the underlying pattern.

Our model treats inferred controllability as a "global" property that generalizes across stimuli. Consequently, it doesn't make sense to plot it separately for different stimuli; by construction it doesn't vary across them.

Finally, please note that there is no second half of each condition block, because the conditions were fully intermixed (see below). Furthermore, there are too few trials per condition to subdivide the conditions so finely. This is a statistical limitation of the experimental design studied here.

In Cavanagh et al. (2013), there are important performance difference between the four conditions as well as between learners and non-learners. Are there differences in model fitting accuracy and in model parameters between conditions or between learners and non-learners? How does the evolution of Pavlovian weight with time differs between these cases? And how does the correlation between frontal theta and Pavlovian weight differ between these cases?

The Cavanagh et al. (2013) paper used a semi-arbitrary post-hoc split of subjects (> or < 65% accuracy on NoGo to Win) to define Learners or Non-Learners, respectively. As stated in that paper, this was only done to simply communicate some rather complex analytic ideas ("This categorical assignment facilitates an intuitive display of performance patterns, but we used continuous measures of learning for all important statistical analyses."). For this reason, here we describe analyses that treat accuracy as a continuous variable.

First, it is indeed the case that people are more accurate when the Pavlovian weight is lower (i.e., inferred controllability is higher). Specifically, the Pavlovian weight is smaller on correct compared to incorrect trials. However, this is not actually very informative, since we know a priori that accuracy will generally be higher when Pavlovian bias is low. This is built into the structure of the model; it's not an empirical discovery. In a similar manner, frontal theta is higher on correct compared to incorrect trials. But again this is not particularly informative, since we've already shown a link between frontal theta and the Pavlovian weight (a true empirical discovery), and the second link between weight and accuracy is a structural property of our model.

Second, we looked at correlations between parameter values and accuracy. We did not find any significant relationships.

From the methods, it is not clear that the four conditions are presented in distinct blocks of trials. In contrast, this is clear from the original papers. I think this should be specified here too. Moreover, I think the results would be different if trials from the four conditions were intermixed, and this should be discussed here. Finally and more importantly, it is not clear to me whether the order between conditions was counterbalanced between subjects or not. This is important since some prior knowledge can be used by subjects and learning can be facilitated during late blocks (especially the fourth one) based on the previously encountered task rules. For instance, in the data of Guitart-Masip et al. (2011), how can one disentangle this effect from the Pavlovian effect to explain the better performance in the fourth block (NoGo to Avoid) than in the third block (NoGo to Win)? Were there significant differences in the initial values of the fitted model between conditions in any of the two datasets? And how did this affect learning and the evolution of the Pavlovian bias?

The conditions were in fact intermixed in both data sets. We now note this explicitly in the Methods.

The authors verified that other model variables, like instrumental and Pavlovian values, do not correlate with the Pavlovian weight, and thus are not potential confounds. But other potential confounds should also be tested here, like choice confidence, reward uncertainty and reward prediction errors, to make sure that the frontal theta power is here only reflecting the suppression of the Pavlovian influence on choice. Along these lines, it has been shown previously that midfrontal theta may relate to reward prediction

errors (Holroyd CB, Krigolson OE, Lee S (2011) Reward positivity elicited by predictive cues. Neuroreport 22:249 –252), to behavioral slowing (Cavanagh JF, Frank MJ, Klein TJ, Allen JJ (2010) Frontal theta links prediction errors to behavioral adaptation in reinforcement learning. Neuroimage 49:3198 –3209), and switching (Cohen MX, Ranganath C (2007) Reinforcement learning signals predict future decisions. J Neurosci 27:371–378; van de Vijver I, Ridderinkhof KR, Cohen MX (2011) Frontal oscillatory dynamics predict feedback learning and action adjustment. J Cogn Neurosci 23:4106–4121). Could the authors here control for these potential predictors of variations in frontal theta power, and whether the Pavlovian bias in the model can account for these effects?

We thank the reviewer for raising the important issue about potential confounds, which we now address on p. 6:

"Our data cannot be explained by alternative theories about midfrontal theta. First, we can rule out an explanation in terms of reward prediction error [Cavanagh et al., 2010, 2012; van Vijver et al., 2011]. At the time of stimulus presentation, the reward prediction error is simply the stimulus value, which is uncorrelated with w ($p=0.29$, signed rank test) as well as with midfrontal theta ($p=0.23$, signed rank test). Second, our data are not adequately explained by choice confidence [Lim et al., 2020]. Although we do not measure subjective choice confidence, we can use as a proxy the expected accuracy under our model. This measure is negatively correlated with the Pavlovian weight ($p<0.001$, signed rank test), as we would expect given that accuracy will tend to be lower under Pavlovian control. However, repeating our quantile comparison using this measure did not reveal a significant relationship between expected accuracy and midfrontal theta power (top vs. bottom quantile: $p=0.14$)."

Incidentally, some of the citations in the reviewer's comment do not actually measure frontal theta, but rather event-related potentials, so their relevance here is unclear.

A broader conceptual point is if and how all these different constructs are related to one another. In our view, the unifying theme is that midfrontal theta reflects need for control. This view is articulated in the Discussion (p. 9):

"More broadly, these results are consistent with the hypothesis that midfrontal theta (and its putative cortical generator in midcingulate / dorsal anterior cingulate cortex) is responsible for computing the ``need for control'' [Cavanagh et al., 2014; Cavanagh & Shackman, 2015] or the ``expected value of control'' [Shenhav et al., 2013]. Controllability is a necessary (though not sufficient) requirement for the exertion of cognitive control to have positive value. From this perspective, it is important to emphasize that we do not see midfrontal theta as exclusively signaling inferred controllability; rather, high inferred controllability signals need for control, which thereby evokes midfrontal theta activity. Other variables that signal need for control, such as reward prediction error [Cavanagh et al., 2010, 2012; van Vijver et al., 2011], can also evoke midfrontal theta activity, without requiring changes in inferred controllability."

About the EEG data, it is a bit unsatisfying to just show a basic correlation between frontal theta power and Pavlovian bias. Canavagh et al. (2013) found that 'interindividual differences in theta power increases in response to Pavlovian conflict (across participants) correlated with intraindividual abilities to use theta (across trials) to overcome Pavlovian biases'. Could the authors here also assess the role of Pavlovian conflict and interindividual differences in the modulation of the correlation between frontal theta power and the Pavlovian bias?

The nature of the modeling in Cavanagh et al. (2013) differs from ours in an important way. In that paper, theta was built into the behavioral model directly. In some sense, it's not terribly surprising that they found a relationship between the theta modulation parameter and individual differences in Pavlovian bias, because the theta modulation parameter was fit to the behavioral data. We find a strong correlation (r=0.63) between average Pavlovian weight (which we already showed tracks theta) and the Pavlovian bias defined by Cavanagh et al. (2013). But this doesn't seem worth reporting, given that the parameters were fit to the behavioral data, so it's not an independent verification of the model.

Our goal in this paper was to exploit the fact that the Bayesian arbitration model makes predictions about variations in Pavlovian bias *within* an individual session. Thus, we feel that focusing on individual differences would be a distraction; it doesn't distinguish the explanatory power of this new model from the explanatory power of earlier models.

The basic analyses of fMRI data presented here are not satisfying either. For instance, it has been shown that vMPFC encodes option values and confidence (M. Lebreton, R. Abitbol, J. Daunizeau, M. Pessiglione (2015) Automatic integration of confidence in the brain valuation signal, Nat. Neurosci., 18(8):1159‑1167; B. De Martino, S. Bobadilla-Suarez, T. Nouguchi, T. Sharot, B. C. Love (2017) Social Information Is Integrated into Value and Confidence Judgments According to Its Reliability, J. Neurosci., 37(25):6066‑6074). Could the authors check for these potential confounds of vMPFC activity, draw links with the model?

Along the lines of our points raised above in connection with the EEG analyses, we expect choice confidence to be inversely related to the Pavlovian weight, if we use expected accuracy as a proxy for confidence. However, this relationship does not attain significance, which we now report on p. 9, along with an analysis of instrumental values:

"*The relationship between Pavlovian weight and vmPFC cannot be explained by choice confidence (again using expected accuracy as a proxy): although we expect expected accuracy to vary inversely with the Pavlovian weight (see above), vmPFC activity did not differ significantly between the top and bottom quantile of expected accuracy (p = 0.08). Nor can the relationship be explained by differences in instrumental values, which are uncorrelated with the Pavlovian weight (median r = 0.02, p = 0.36, signed rank test).*"

Some methodological information is missing. For instance, is the reported frontal theta power phase-locked or not, and what does this imply in terms of interpretation? In the fMRI data, to which events of the task are the IFG and vMPFC responses shown?

Thanks for pointing out this missing information. As we now clarify in the Methods, frontal theta was computed as total spectral power (both phase-locked and non-phase-locked). We have also added text to the Discussion that highlights the potential for future work on this question (p. 10):

"Finally, in accordance with most prior analyses of midfrontal theta, we focused only on total theta power, neglecting any distinctions between phase-locked and non-phase-locked activity. However, several lines of research suggest that these two components of oscillatory activity carry different information [Hajihosseini & Holroyd, 2013; Cohen & Donner, 2013]. Distinguishing them may therefore support a finer-grained dissection of computational function."

With regard to the fMRI data, our analyses are restricted to stimulus-locked activity, as described in the Methods. We have now made this more explicit in the Results section as well.

Typos

Page 6, differed fom 0 -> from 0.

Fixed.

Pages 7 and 11, please fix three occurrences of 'NoGo¿Go'.

Fixed.
