# Supplementary material for: Neural signatures of arbitration between Pavlovian and instrumental action selection
Source: PLoS Comput Biol. 2021 Feb 10;17(2):e1008553. doi: 10.1371/journal.pcbi.1008553 (PMC7901778; doi:10.1371/journal.pcbi.1008553)
Supplement: Attachment — Submitted filename: response.pdf [file pcbi.1008553.s007.pdf]

Dear Dr. Marinazzo,

Thank you for giving us another opportunity to revise our manuscript. Below we respond to each comment point-by-point.

Best,

Sam Gershman

Reviewer 1

I really like the author's modelling of the neural data, and agree that their project makes sense as a follow up and consolidation of the earlier modelling of behavioural data. The results of the analysis of midfrontal theta and VMPFC effects are novel and would be useful to others who work in this field.

I regret that in my view, the authors' response to my critique was not sufficiently robust. Put simply, the original manuscript was motivated by behavioural findings X and Y; therefore, there need to be a bit more reconning, when it is pointed out that the findings are actually ~X and ~Y.

While I agree with the authors that the go-no-go task could, in principle, rely on different principles than the PIT task, and it is absolutely right to point out any links between these tasks should be tested empirically, the logic of the model is based on the hypothesis that the task taps generic Pavlovian and Instrumental processes. This is, indeed, how the abstract and introduction are written.

Based on the reviewer's previous comments, which pointed out correctly that we had overstated the generality of our framework, in our last revision we removed the discussion of PIT from the Introduction, and placed this as a direction for future work at the end of the Discussion. We do not claim anywhere in the Introduction or Abstract that the Go/NoGo task taps "generic Pavlovian and instrumental processes". Nor do we claim that the model is a generic model of all interactions between these processes. Nor do we state anywhere that the logic of the model is based on such a hypothesis.

Therefore, when they say that the "Pavlovian weight can also be interpreted as the subjective degree of belief in an uncontrollable environment", it makes sense for readers to reflect on situations where an agent is trained less well in the instrumental task, and therefore, their belief in the controllability of the environment should be lower. When the same agent is given an opportunity to behave in a way that can reflect both instrumental and Pavlovian biases, then if I had understood the model correctly, the Pavlovian biases

should be stronger when instrumental training was extensive, and weaker when instrumental training was less extensive. Yet in this situation, during a PIT test, the empirical finding is exactly the opposite. As the PIT situation is the best-studied example of the interaction of Pavlovian and instrumental processes, readers are likely to have this example in mind.

To clarify, Pavlovian bias will be stronger in our model when instrumental training is *less* extensive. However, we wish to emphasize again that we have not actually modeled PIT anywhere in the paper. We can see why this is a natural inference to make, but until we undertake a systematic study of this question, it doesn't make sense to draw such inferences. This is why we chose to focus more narrowly on the Go/NoGo task, for which we have been able to build a systematic case for our model.

The work and the data remain interesting if the model can only explain the go-no-go task; or tasks where the stimuli are spaced in particular ways; but this needs to be stated clearly in the introduction with the boundaries laid out.

In the Introduction, when we first introduce the Go/NoGo task, we now state: *"We focus on this task in the present paper, while acknowledging that our conclusions may not generalize to other forms of Pavlovian bias, such as in Pavlovian-instrumental transfer paradigms."*

At the end of the Discussion, we state: *"While our work is partly motivated by studies of Pavlovian-instrumental transfer [Holmes et al., 2010], it is still an open empirical question to what extent that phenomenon is related to Pavlovian biases in Go/NoGo performance. It is also an open theoretical question to what extent the kind of Bayesian arbitration model proposed here can provide a comprehensive account of Pavlovian-instrumental transfer."*

This is a statement of the boundary conditions. We don't want to state that the model *can't* explain PIT until we actually undertake a systematic study of that question, which would be a completely new paper.

Minor comments

1."At the time of stimulus presentation, the reward prediction error is simply the stimulus value – " Would participants not update the reward prediction based on their performance on previous trials, and the reward offered on previous trials, such that the reward they can predict at the start of the new trial varies somewhat?

We have clarified this: *"At the time of stimulus presentation, the reward prediction error is simply the estimate of stimulus value (a recency-weighted average of past prediction errors)"*

2.In a number of places, the authors rule out alternative interpretation of the data by referring to null results of a signed rank test – e.g. three places on p. 6 and one place at the top of p. 7. This is a worry, because while the sample may have been powered for the

purpose of the original study, it may not have been sufficiently large to allow the detection of the effects the authors refer to here (e.g. if it was powered to detect difference between means, it may not be large enough to detect a significant correlation). Please could the authors state the statistical power they had in every case they refer to a null result, e.g. "the median correlation never significantly differed from 0, although we only had power to detect effects that are medium in magnitude, or higher".

Thanks for this suggestion. We think an arguably better way to support the null hypothesis is to report Bayesian analyses, which we now do.

"To evaluate the positive evidence for the null hypothesis (correlation of 0), we also carried out Bayesian t-tests [Rouder et al., 2009] on the Fisher z-transformed correlations, finding that the Bayes factors consistently favored the null hypothesis (ranging between 2 and 5)." (p. 6)

"At the time of stimulus presentation, the reward prediction error is simply the estimate of stimulus value (a recency-weighted average of past prediction errors), which is uncorrelated with w (p=0.29; signed rank test; Bayes factor favoring the null: 2.36, Bayesian t-test applied to Fisher z-transformed correlations) as well as with midfrontal theta (p=0.23, signed rank test; Bayes factor favoring the null: 2.62)." (p. 7)

"Nor can the relationship be explained by differences in instrumental values, which are uncorrelated with the Pavlovian weight (median r = 0.02, p=0.36, signed rank test; Bayes factor favoring the null: 3.30, Bayesian t-test applied to Fisher z-transformed correlations)." (p. 8)
